# A Cross-Sectional Pharmacoepidemiological Study of the Utilization Pattern of Pre-Anesthetic Medications in Major Surgical Procedures in a Tertiary Care Hospital

**DOI:** 10.7759/cureus.1344

**Published:** 2017-06-13

**Authors:** Madhuri Kulkarni, Anant Patil

**Affiliations:** 1 Pharmacology, Govt Medical College, Aurangabad, Maharashtra, India; 2 Pharmacology, Bharati Vidyapeeth Deemed University Dental College & Hospital, Navi Mumbai

**Keywords:** drug utilization, pre-anesthetic medications, surgical procedures

## Abstract

**Objective:**

A study of the utilization pattern of pre-anesthetic medications in major surgical procedures.

**Material and methods:**

A cross-sectional, pharmacoepidemiological study was conducted among patients undergoing surgical procedures in routine or emergency conditions under general anesthesia. The utilization of pre-anesthetic medicines in all patients was reported.

**Results:**

A total of 110 patients (mean age - 42.36 years; 57.27% males) were enrolled. The major (>10%) indications for surgery were carcinoma/tumor - 25 (22.73%), perforation peritonitis - 20 (18.18%), and intestinal obstruction - 11 (10%). All patients received ranitidine plus metoclopramide. Injections of ondansetron, midazolam, pentazocin, anticholinergic agents, and fentanyl were used in 103 (93.64%), 103 (93.64%), 92 (83.64%), 84 (76.36%), and 23 (20.91%) patients, respectively. The dosage of ondansetron was 4 mg in 95 patients (86.36%), while 89.09% patients received 1 mg of midazolam. In 86 patients (78.18%), pentazocine was used in a 15 mg dose. Among the anticholinergic agents, glycopyrrolate was used in 76 patients (69.09%). Glycopyrrolate was used in a 0.2 mg dose in 74 patients (97.37%). The major indications for the use of fentanyl were carcinoma - 8 (34.8%), perforation peritonitis - 4 (17.4%), and surgery for intestinal obstruction - 3 (13%).

**Conclusion:**

The administration of ondansetron, midazolam, and pentazocin is very common (>80% patients) as pre-anesthetic medication. Glycopyrrolate was the preferred anticholinergic agent. In cancer patients, the use of fentanyl is common.

## Introduction

Drug utilization studies are usually conducted to understand medicine usage patterns in a hospital setting, to facilitate improvements, and to provide an uninterrupted supply of commonly used medicines. The research also helps minimize the risk of adverse events and drug interactions, which can contribute to better therapeutic outcomes. Regular prescription audits also help in evaluating and, if necessary, in suggesting changes in prescribing practices to facilitate rational and cost-effective medical care.

Major concerns during surgical procedures include anxiety among patients, post-operative pain, post-operative nausea and vomiting, and the risk of aspiration pneumonitis. Pre-anesthetic medicines are generally given to avoid the adverse events associated with general anesthesia, facilitate surgery, and reduce the risk of post-operative complications [1]. The agents used as pre-anesthetic medications include agents to reduce gastric acidity, benzodiazepines [2-3], anticholinergic agents [4-5], antiemetics [6], pentazocine [7], and opioid analgesics. Pre-anesthetic agents are given about half an hour to one hour before an anesthetic agent, with the objective of making anesthesia safer and more agreeable to the patient. Pre-anesthetic medications are used depending on the patient’s clinical status and the type and duration of operation. Drug utilization studies on pre-anesthetic medicines in India are limited. This study was conducted to understand the prescription and utilization patterns of pre-anesthetic medications for different surgical procedures.

## Materials and methods

In this cross-sectional, pharmacoepidemiological, and observational study, patients of both sexes and of all age groups receiving pre-anesthetic medication for surgical procedures in routine or emergency conditions were included.

The prescription and utilization of pre-anesthetic medicines in all patients undergoing surgeries under general anesthesia for various indications were recorded during a six-month period. After recording the patient’s demographic data, the indications of surgery and the details of the pre-anesthetic medications used were recorded. The study was initiated after receiving approval from the institutional ethics committee.

### Statistical analysis

Categorical data are presented as numbers and percentages, while continuous data are presented as mean and standard deviations (SD).

## Results

A total of 110 patients (57.27% males) were enrolled in this study. The mean age of study participants was 42.36 (+18.36) years. The minimum age of study participants was 3 days and the maximum was 76 years (Table 1).

**Table 1:** Baseline characteristics

Parameter	Result (n=110)
Mean age (SD) in years	42.36 (+18.36)
Range	3 days to 76 years
Male n (%)	63 (57.27%)
Female n (%)	47 (42.73%)

The major (>10%) indications for surgery included carcinoma/tumor - 25 (22.73%), perforation peritonitis - 20 (18.18%), and intestinal obstruction - 11 (10%). The number and percentages of indications for surgeries are shown in Table 2.

**Table 2:** Indications for surgery

Condition	N (%)	Condition	N (%)	Condition	N (%)
Carcinoma/tumor	25 (21.82%)	Congenital diaphragmatic hernia	2 (1.82%)	Fibroid with severe menorrhagia	1 (0.91%)
Perforation peritonitis	20 (18.18%)	Chronic appendicitis	2 (1.82%)	Duodenal perforation with peritonitis	1 (0.91%)
Intestinal obstruction	11 (10%)	Chronic tonsillitis	2 (1.82%)	Right deviated nasal septum	1 (0.91%)
Lower (uterine) segment caesarean section	8 (7.27%)	Small bowel gangrene with peritonitis	1 (0.91%)	Swelling on the right side of the neck	1 (0.91%)
Appendix perforation	5 (4.55%)	Chronic tonsillits	1 (0.91%)	Chronic dacryocystitis	1 (0.91%)
Chronic suppurative otitis media	4 (3.64%)	Peritonitis with small bowel obstruction	1 (0.91%)	Severe postpartum haemorrhage (PPH)	1 (0.91%)
Blunt trauma to the abdomen	4 (3.64%)	Left frontoparieta subdural hematoma	1 (0.91%)	Pseudoarthrosis	1 (0.91%)
Fracture (hip/humerus)	3 (2.73%)	Inversion of testes	1 (0.91%)	Bowel gangrene	1 (0.91%)
Chronic pancreatitis	2 (1.82%)	Subdural bleed	1 (0.91%)	Anterior dislocation of shoulder	1 (0.91%)
Gastroschisis	2 (1.82%)	Gangrene stump	1 (0.91%)	Right ear perforation	1 (0.91%)
Penetrating injury	2 (1.82%)	Head injury	1 (0.91%)		

**Figure 1:**
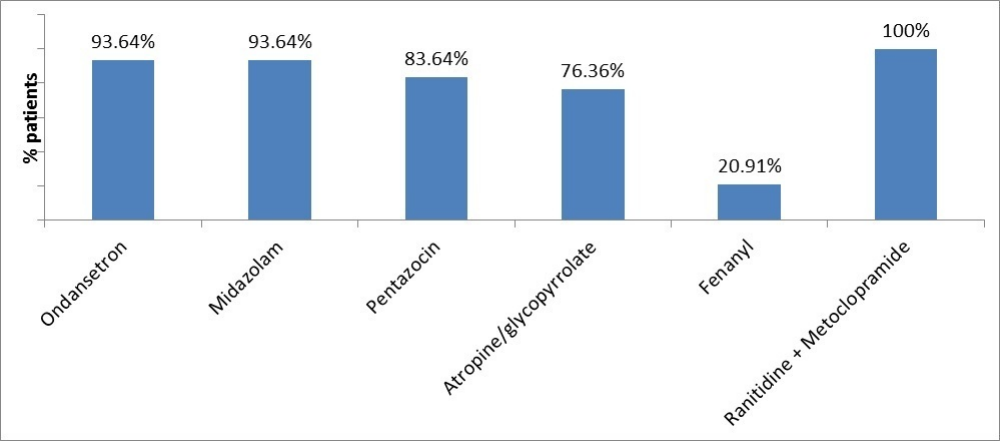
Pre-anesthetic medications used in the study (n=110)

Figure 1 shows a list of pre-anesthetic medications. All patients received ranitidine plus metoclopramide. Ondansetron and midazolam injections were used in 103 (93.64%) patients each. Pentazocin and anticholinergic agents were used in 92 (83.64%) and 84 (76.36%) patients, respectively. Fentanyl injection was administered in only 23 (20.91%) patients.

A 4 mg dose of ondansetron was administered to 95 patients (86.36%). The number of patients receiving 2 mg and 1 mg ondansetron were three (2.73%).

**Figure 2:**
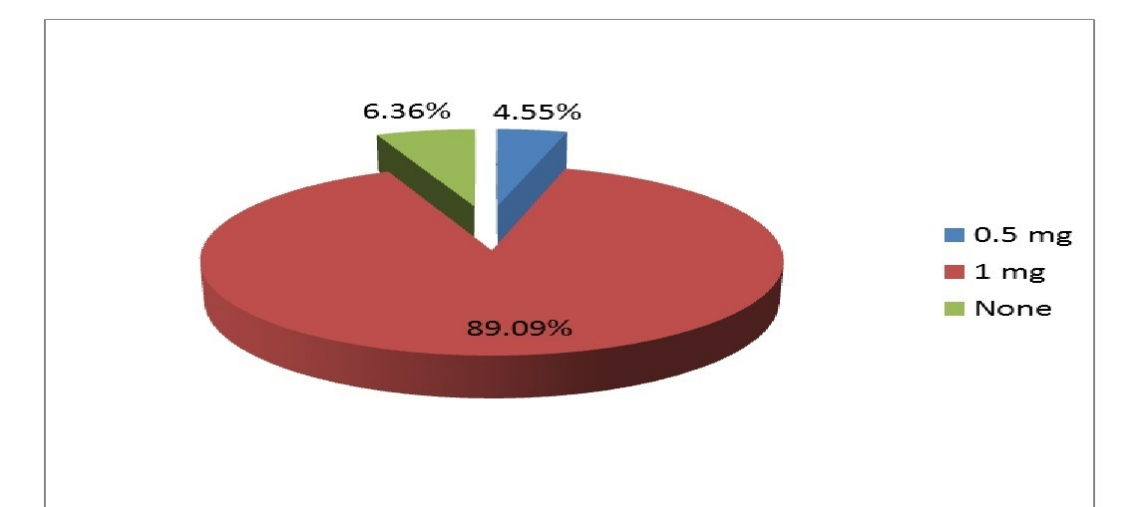
Use of midazolam in the study

A total of 103 (93.64%) patients received midazolam, while seven (6.36%) patients did not receive it (Figure 2). Overall, 89.09% patients received a 1 mg dose of midazolam, while 0.5 mg was used in 4.55% patients (Figure 2).

**Figure 3:**
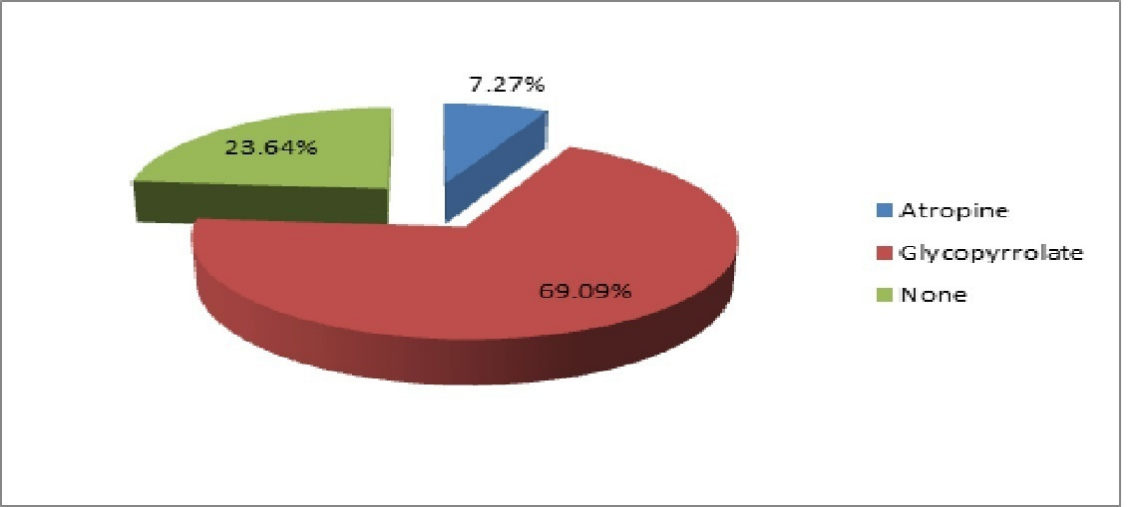
Usage of anticholinergic agents as pre-anesthetic medication

In 86 patients (78.18%), a 15 mg dose of pentazocine was used, while in other patients, a smaller dose was used. Among the anticholinergic agents, glycopyrrolate was used in 76 patients (69.09%), while in eight patients (7.27%), atropine was used. In 26 patients (23.64%), no anticholinergic agent was used (Figure 3).

Out of the eight patients to whom atropine was administered, a 100 mcg dose was used in one patient, while in other patients, a smaller dose was used.

Glycopyrrolate was administered as a 0.2 mg dose to 74 patients (97.37%), while 0.1 mg was administered to two patients (2.63%).

Fentanyl was used in 23 patients. The major indications for the use of fentanyl were carcinoma - 8 (34.8%), perforation peritonitis - 4 (17.4%), and surgery for intestinal obstruction - 3 (13%). Other indications for the use of fentanyl are given in Table 3.

**Table 3:** Indications for the use of fentanyl (n=23)

Indication	N (%)
Carcinoma	8 (34.8%)
Perforation peritonitis	4 (17.4%)
Intestinal obstruction	3 (13%)
Ruptured appendix	1 (4.3%)
Bowel gangrene	1 (4.3%)
Gastroschisis	1 (4.3%)
Caesarean section	1 (4.3%)
Acute appendicitis with perforation	1 (4.3%)
Primigravida with obstructed labor	1 (4.3%)
Chronic suppurative otitis media	1 (4.3%)
Chronic dacryocystitis	1 (4.3%)

Out of eight patients to whom fentanyl was administered, three (35.5%) had cancer of the buccal mucosa, while malignant ovarian tumor, cancer of the tongue, lip, and esophagus, and cholangiocarcinoma were present in one patient each (Table 4).

**Table 4:** Types of cancer in which fentanyl was used (n=8)

Indication	N (%)
Cancer of buccal mucosa	3 (35.5%)
Malignant ovarian tumor	1 (12.5%)
Cancer of tongue	1 (12.5%)
Cancer of lip	1 (12.5%)
Cancer of esophagus	1 (12.5%)
Cholangiocarcinoma	1 (12.5%)

## Discussion

Drug utilization studies are important for scientific as well as administrative purposes in large hospital settings, especially tertiary care centers. Significant insights into the use of medicines, indications, side effects, and drug interactions can be obtained from such studies. There are limited data from Indian settings showing trends in the usage of pre-anesthetic medications in patients undergoing surgeries under general anesthesia. We performed a cross-sectional study in a tertiary healthcare setup to help understand the utilization of pre-anesthetic medicines.

Nausea and vomiting are one of the major concerns in patients undergoing surgery. The concern is because of two reasons: One, the morbidity it causes in patients, and two, the risk of surgical complications [6]. Several drugs, including antihistaminics, anticholinergics, dopamine antagonists, and phenothiazine derivatives, have been used in the management of post-operative nausea and vomiting. Unwanted side effects limit the use of most of these drugs [8]. The introduction of 5-HT3 receptor antagonists is one of the major milestones in antiemetic therapy [9-10]. These agents are significantly effective in the prevention of post-operative nausea and vomiting. Ondansetron, the first agent introduced in this class, is devoid of any significant effect on other receptors (e.g., dopamine, histamine, or sympathetic/parasympathetic receptors) apart from 5-HT3 [9].

In our study, two of the most commonly used antiemetic agents were ondansetron and metoclopramide. The prophylactic administration of intravenous (IV) ranitidine and metoclopramide reduces the volume of gastric content and increases the gastric pH, reducing the risk of aspiration pneumonitis in patients receiving anesthesia [11]. In our study, all patients received metoclopramide plus ranitidine injections.

Benzodiazepines are used to reduce anxiety in patients undergoing surgery. Diazepam was the preferred agent for this purpose for several years. However, today, midazolam is preferred over diazepam because of its higher potency, faster onset of action, and shorter duration of action [2]. A study has shown that in patients between 60 and 69 years of age, 2 or 3 mg of intramuscular midazolam is an effective pre-anesthetic medication and does not cause severe drowsiness. However, in patients aged 70 years and above, it might cause severe drowsiness [3]. In our study, the most commonly used dose of midazolam was 1 mg. In this dose, midazolam was well-tolerated without significant concerns of drowsiness.

Intravenous pentazocine can be used preoperativelyto reduce intraoperative hemodynamic changes, pain following surgery, and fentanyl-induced cough [7-12]. The combination of pentazocin-phenergan-midazolam is better than the ketamine-midazolam combination along with local anesthesia in terms of hemodynamic changes and provides a longer duration of analgesia. Similarly, patient satisfaction with the triple drug combination is also better than with the ketamine-midazolam combination for surgeries performed under monitored anesthesia care [13]. In our study, the use of pentazocine and midazolam was very common, but the use of phenergan was not.

Anticholinergic agents are routinely prescribed pre-anesthetic medications. Atropine and glycopyrrolate are two well-studied pre-anesthetic anticholinergic agents. Glycopyrrolate is more potent than atropine, and it consequently needs a lower dose. The other advantages of glycopyrrolate include a more-stable response in the cardiovascular system, less risk of bradycardia, and better control of oropharyngeal secretions during reversal [4]. 

A comparative study showed a similar effect to that of ondansetron when using glycopyrrolate for nausea and vomiting during a cesarean section [6].

Even in children, glycopyrrolate is the anticholinergic agent most commonly used as pre-anesthetic medication [5-6]. Consistent with findings in the literature, glycopyrrolate was the most commonly used anticholinergic agent in our study. The use of glycopyrrolate was almost 10 times more common than that of atropine in the present study.

Pain is one of the major concerns in patients with cancer, apart from other negative consequences on patients and relatives. Pain management is individualized based on factors including the type of cancer, available medicine, the side-effects and profile of the drug, and so on [14]. A short-acting opioid, such as fentanyl, is preferred over long-acting opioids during surgical procedures because of the higher risk of postoperative adverse effects, such as respiratory depression, with long-acting agents. Similarly, in a low dose, fentanyl can decrease the hemodynamic response to tracheal intubation [15]. In our study, fentanyl was used in 23 patients. Out of those patients who received fentanyl, cancer was the most common indication for its use. In almost one-third of the patients, fentanyl was used for pain control in cancer patients. Cancer of the buccal mucosa was the most common indication among all cancers.

Our study has some limitations. The small sample size, single-center data, and cross-sectional design limit the generalization of our findings to all surgical procedures. We did not evaluate the differences in drug utilization with different induction and maintenance agents for anesthesia. Similarly, the incidence of side effects and patient satisfaction was not evaluated in the study. With these limitations, we suggest that the findings of our study be carefully extrapolated.

## Conclusions

Ondansetron, midazolam, and pentazocin are the main (>80% patients) pre-anesthetic medications used for major surgeries in a tertiary care hospital. The usage of glycopyrrolate is also common and it is the preferred anticholinergic agent. Fentanyl is a commonly used pre-anesthetic medicine in patients with cancer. 
